# Associations between Total Cerebral Blood Flow and Age Related Changes of the Brain

**DOI:** 10.1371/journal.pone.0009825

**Published:** 2010-03-23

**Authors:** Adriaan C. G. M. van Es, Jeroen van der Grond, V. Hester ten Dam, Anton J. M. de Craen, Gerard J. Blauw, Rudi G. J. Westendorp, Faiza Admiraal-Behloul, Mark A. van Buchem, for the PROSPER Study Group

**Affiliations:** 1 Department of Radiology, Leiden University Medical Center, Leiden, The Netherlands; 2 Section of Gerontology and Geriatrics, Department of General Internal Medicine, Leiden University Medical Center, Leiden, The Netherlands; 3 Section of Image Processing (LKEB), Department of Radiology, Leiden University Medical Center, Leiden, The Netherlands; New York State Institute for Basic Research, United States of America

## Abstract

**Background and Purpose:**

Although total cerebral blood flow (tCBF) is known to be related to age, less is known regarding the associations between tCBF and the morphologic changes of the brain accompanying cerebral aging. The purpose of this study was to investigate whether total cerebral blood flow (tCBF) is related to white matter hyperintensity (WMH) volume and/or cerebral atrophy. Furthermore, we investigate whether tCBF should be expressed in mL/min, as was done in all previous MR studies, or in mL/100 mL/min, which yielded good results in precious SPECT, PET and perfusion MRI studies investigating regional cerebral blood flow.

**Materials and Methods:**

Patients were included from the nested MRI sub-study of the PROSPER study. Dual fast spin echo and FLAIR images were obtained in all patients. In addition, single slice phase contrast MR angiography was used for flow measurements in the internal carotids and vertebral arteries. tCBF was expressed in both mL/min and mL/100 mL/min.

**Results:**

We found a significant correlation between tCBF in mL/min and both age (*r* = −.124; *p* = *p*≤.001) and parenchymal volume (*r* = 0.430; *p*≤.001). We found no association between tCBF in mL/min and %-atrophy (*r* = −.077; *p* = .103) or total WMH volume (*r* = −.069; *p* = .148). When tCBF was expressed in mL/100 mL/min the correlation between tCBF and age was no longer found (*r* = −.001; *p* = .985). Multivariate regression analyses corrected for age showed a significant correlation between tCBF in mL/100 mL/min and WMH volume (*r* = −.106; *p* = .044). No significant association between tCBF in mL/100 mL/min and %-atrophy was found.

**Conclusion:**

From this study we conclude that, when evaluating tCBF alterations due to various pathologies, tCBF should in mL/100 mL/min instead of mL/min. Furthermore, changes or differences in WMH volume should be accounted for.

## Introduction

Determination of total cerebral (tCBF) blood flow to the brain, by measuring flow in the internal carotid arteries (ICA) and posterior circulation has been applied in patients with obstructive disease of the ICA or posterior circulation[1], [2], arteriovenous malformations[3], acute neurotrauma[4], cerebral ischemia[5], and the evaluation of vascular interventions such as bypass surgery[6] or carotid endarterectomy[7]. In these studies tCBF was expressed in mL/min, regardless of the volume of the supplied brain. However, for interpretation of the data, Buijs et al. have published a flow decrease of 4.8 ml/min per year in a normal population[8]. In addition, Hendrikse and coworkers have shown that the distribution of flow in the brain is strongly influenced by the anatomy of the circle of Willis[9]. Determination of flow to the vessels that supply the brain seems especially useful in subjects in whom it may be expected that the volume flow is getting too low to maintain normal cerebral blood flow and function such as elderly subjects or patients suffering from Alzheimer's disease, diabetes or atherosclerosis.

White matter hyperintensities (WMHs) and cerebral atrophy are common findings in the aging population and their prevalence increases with age[10]–[12]. On T2-weighted Magnetic Resonance Imaging (MRI) WMHs are seen as patchy or diffuse areas of hyperintensity. Clinically they have been associated with gait disturbance[13], cognitive impairment[14], [15], mood disorder[16] and dementia[17]. Changes in CBF might also reflect cerebral changes such as atrophy. In addition to an increased WMH load, cerebral atrophy is a common finding in the elderly and a manifestation of neuronal degeneration contributing to cognitive decline and dementia[18], [19].

Low regional cerebral blood flow is one of the processes thought to underlie the development of both WMHs and atrophy as strong associations with vascular risk factors exist[10], [20]–[24]. Moreover, declining blood pressure has also been associated with global brain atrophy[25]. The associations between flow and WMHs have been investigated in positron emission topography (PET), single photon emission topography (SPECT) and perfusion weighted MRI studies, showing a relationship between low regional cerebral blood flow (rCBF) and WMHs in small populations[26]–[33]. All these studies expressed the rCBF in mL/100mL/min. Therefore, contrary to previous MR studies, these flow measurements are not influenced by potential influence of the volume of the provided (part of the) brain.

Despite this evidence for an important role of the cerebral blood supply in the development of both WMHs and atrophy, only one study has investigated the association between tCBF and WMHs[34]. This study found that patients in the quartile with the highest tCBF had a lower number and severity of WMHs suggesting an association between tCBF and the presence of WMHs. However this study has two major limitations. First, WMHs were scored quantitatively and analyzed in quartiles. Second, similar to previous MR studies, the tCBF was expressed as mL/min and therefore the effect of parenchyma volume was not taken into account.

The aim of this study is two-fold: 1) to investigate whether tCBF is related to WMHs volume and/or cerebral atrophy and 2) whether these associations are influenced by expressing tCBF in mL/100 mL/min instead of mL/min.

## Methods

### Ethics Statement

The PROSPER study had ethics review board approval of all locations and written informed consent of all participants. In addition, the Leiden University Medical Center institutional ethics review board approved the protocol for the prospective MR study and subsequent retrospective analyses. Moreover, all participants gave written informed consent. Participants of this MR study also agreed with future retrospective analysis of their MR data for research purposes.

### Patients

Patients were included from the nested MRI substudy of the PROspective Study of Pravastatin in the Elderly at Risk (PROSPER). Inclusion criteria for this study were: men or women aged 70–82 years; total cholesterol 4.0–9.0 mmol/L; stroke, transient ischemic attack, myocardial infarction, arterial surgery, or amputation for vascular disease >6 months before study entry; ≥1 of the following risk factors for vascular disease: current smoker; hypertension, currently receiving drug treatment; known diabetes mellitus or fastening blood glucose >7 mmol/L. Exclusion criteria have been described in detail elsewhere[35]. All subjects had a history of, or were at increased risk for, vascular disease. In total, 464 subjects underwent MRI including flow measurements. 17 patients dropped out because flow measurements of the ICAs or vertebral arteries or segmentation of the intracranial structures failed because of technical problems. Patient characteristics are shown in *Table 1*.

**Table 1:** Demographic and clinical characteristics.

Continuous variates	means (SD)
Age, y	75 (3)
Systolic blood pressure, mm Hg	158 (23)
Diastolic blood pressure, mm Hg	86 (11)
Total cholesterol, mmol/L	5.8 (0.8)
LDL cholesterol mmol/L	3.9 (0.7)
HDL cholesterol, mmol/L	1.2 (0.3)
Triglycerides, mmol/L	1.5 (0.7)
Total WMH volume, ml	5.2 (9.6)
Categorical variates	n (%)
Male gender	262 (56)
Current smoker	96 (21)
History of diabetes	76 (16)
History of hypertension	293 (63)
History of myocard infarction	56 (12)
History of stroke or TIA	75 (16)
History of any vascular disease	202 (44)
MRI stroke	178 (38)

LDL  =  Low-density lipoprotein.

HDL  =  High-density lipoprotein.

WMH  =  White matter hyperintensity.

n  =  number of patients.

%  =  percentage of patients.

### MRI

**Figure 1:**
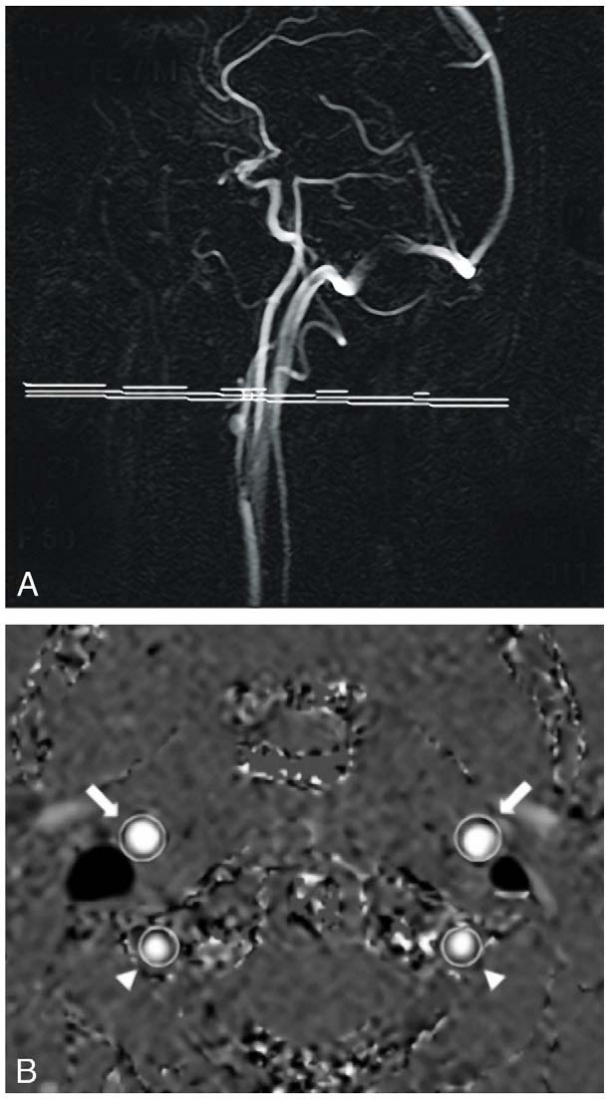
Single slice phase contrast MR angiography. Saggital MRA scout image of the main brain feeding arteries (**a**). The white slab represents the position of the transverse angiographic phase image. This image shows the carotid arteries (arrows) and vertebral arteries (arrowheads) over which flow was measured (**b**).

All imaging was performed on an MR system operating at a field strength of 1.5 Tesla (Philips Medical Systems, Best, The Netherlands). Dual fast spin echo (TE = 27/120 msec; TR = 3000 msec; flip angle = 90°; section thickness = 3 mm; number of sections = 48; no section gap; whole brain coverage; FOV = 220; scan matrix = 256×204; nsa = 1) and fluid-attenuated inversion recovery (FLAIR) (TE = 100 msec; TR = 8000 msec; flip angle = 90°; section thickness = 3 mm; sections = 48; no section gap; whole brain coverage; FOV = 220; scan matrix = 256×204, nsa = 1) images were obtained from all subjects. In addition, single slice phase contrast MR angiography (TR/TE = 16/9 msec; flip angle = 7.5°; slice thickness = 5 mm; FOV = 250; RFOV = 75%; scan percentage = 80%; matrix = 256; 8 signal averages) with a velocity encoding of 100 cm/second was used for flow measurements[36]. The scans were performed in a plane perpendicular to the left and right internal carotid artery and the vertebral arteries, at the level of the vertical segment of the petrous portion of the internal carotid artery (*Figure 1* *)*.

### Image Analysis

**Figure 2:**
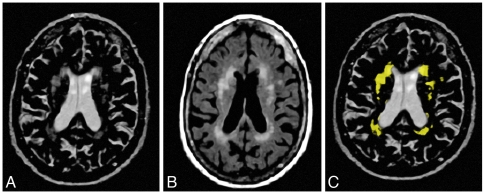
The segmentation process using SNIPER (Software for Neuro-Image Processing in Experimental Research). T2-weighted image (**a**) and FLAIR image (**b**). Result of automated segmentation process based on both sequences (**c**).

WMHs, intracranial volume, and brain parenchyma were assessed semiautomatically. That is, segmentations of WMHs, intracranial volume, and brain parenchyma were generated automatically using Software for Neuro-Image Processing in Experimental Research (SNIPER), an in-house developed program for image processing (*Figure 2*.). The WMH volume was calculated automatically[37]. Infarcted areas were counted being CSF. All measurements were performed blinded to subject identity, age and sex. Atrophy was calculated using the formula: atrophy (%)  =  ((intracranial volume – parenchymal volume)/intracranial volume) ×100%. For tCBF assessment, images were analyzed using the software package FLOW®[38]. For this analysis a region of interest (ROI) was manually drawn around the left and right internal carotid and the right and left vertebral arteries in the magnitude image by one observer (ACGMvE). The flow in these four vessels was summed giving the tCBF in mL/min. TCBF was also expressed in mL blood per 100 mL of brain parenchyma per min (mL/100 mL/min).

### Statistical analysis

SPSS for Windows (release 12.0; SPSS, Chicago, IL) was used for data analysis. Linear regression analysis was used to investigate correlations between parameters. Age was entered as an independent variable as previous studies have reported an inverse correlation between tCBF and age[8], [9]. The level of significance was set at *p<0.05*.

## Results

**Figure 3:**
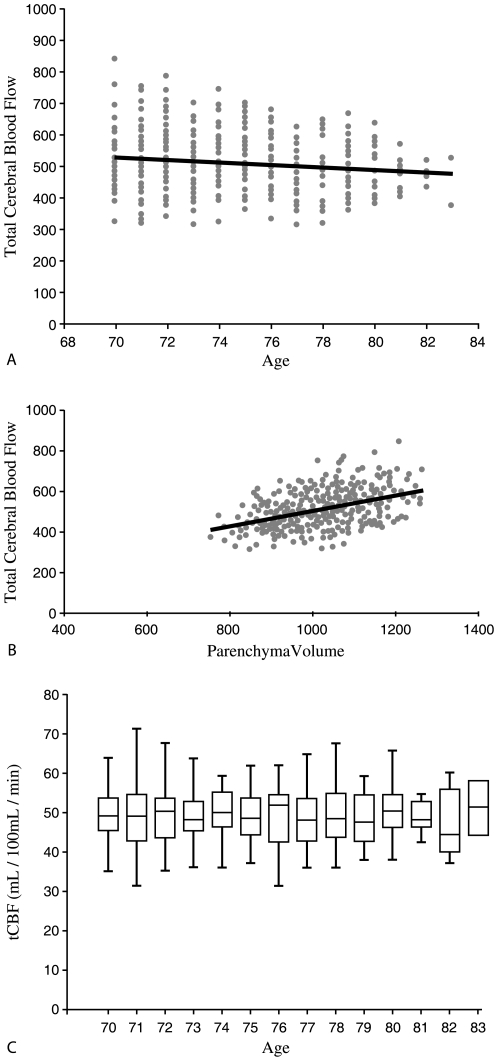
Association of tCBF with age and parenchyma volume. Scatterplot of tCBF (mL/sec) versus age (yrs) (*r* = –.124; *p* = 0.008) (**a**) and parenchymal volume (mL) (*r* = 0.430; *p*≤.001) (**b**). Box-and-whisker plot of average tCBF (mL/100 mL/min) found for each age (yrs) (**c**).

The MRI characteristics of the subjects included in this study are shown in *Table 2*. In this study an inverse correlation between age and tCBF in mL/min was found (*r* = –.124; *p* = 0.008; *Figure 3a*). This corresponds with an annual flow decrease of 6.2 mL. *Figure 3b* shows the association between tCBF in mL/min and parenchymal volume, indicating a significant correlation (*r* = 0.430; *p*≤.001) between the two. When tCBF was expressed in mL/100 mL/min the correlation between tCBF and age was no longer found (*r* = –.001; *p* = .985) (*Figure 3c*). The volume flow (mL/min) in the individual vessels contributing to the tCBF did not show an association with age. However, significant correlations with parenchymal volume were found for both left (*r* = .114; *p* = .016) and right (*r* = .119; *p* = .012) vertebral artery and both left (*r* = .293; *p*≤.001) and right (*r* = .284; *p*≤.001) internal carotid artery.

**Table 2:** MR characteristics.

	mean	SD	minimum	maximum
tCBF in mL/min	510	87.4	315	846
tCBF in mL/100 mL/min	50.4	7.8	30.9	74.3
Parenchymal vol. (mL)	1016	98.7	752	1259
Atrophy (%)	27.3	3.2	15.6	38.4
tot. WMH vol. (mL)	7.1	10.6	0	76.2

tCBF  =  Total cerebral blood flow.

WMH  =  White matter hyperintensity.

**Figure 4:**
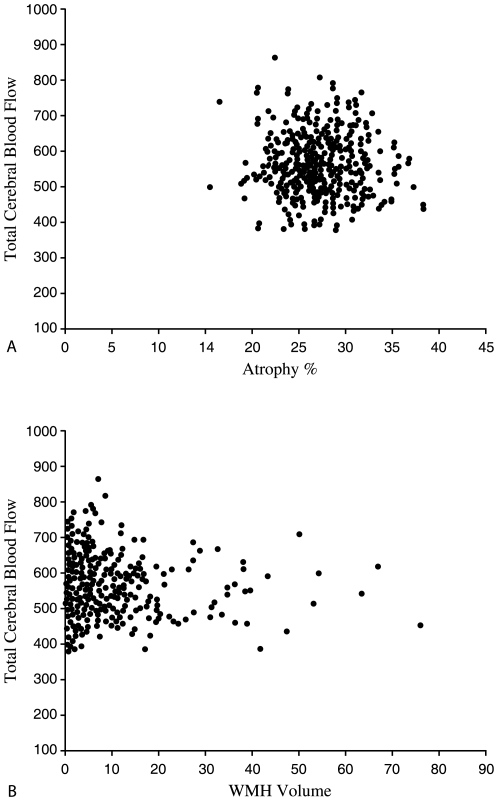
Association of tCBF (in mL/min) with atrophy and WMH volume. Scatterplot of tCBF (mL/sec) versus atrophy (%) (*r* = –.077; *p* = .103) (**a**) and total WMH volume (mL) (*r* = –.069; *p* = .148) (**b**). Atrophy was defined by the following equation: intracranial volume - parenchyma volume/intracranial volume. Therefore, number reflects the percentage of intracranial volume occupied by cerebrospinal fluid.

**Figure 5:**
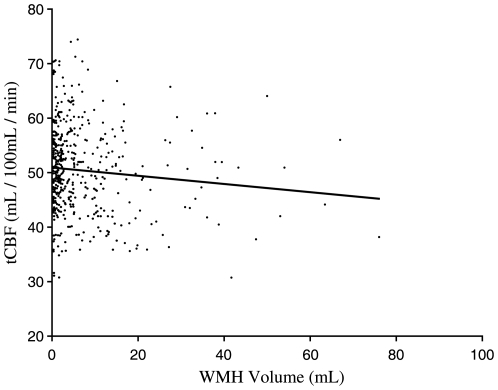
Association of tCBF (mL/100 mL/min) with WMH volume. Scatterplot of tCBF (mL/100 mL/min) versus WMH volume (mL).

We found no association between tCBF in mL/min and %-atrophy (*r* = –.077; *p* = .103) or total WMH volume (*r* = –.069; *p* = .148) (*Figure 4a &* *4b*). TCBF in mL/100 mL/min was associated with WMH volume (*r* = –.106; *p* = .044) (*Figure 5*). No significant association between tCBF in mL/100 mL/min and %-atrophy was found. Furthermore, no association between parenchyma volume and WMH volume existed (*r* = .05; *p* = .317).

## Discussion

The findings of the present study are fourfold. First, the most important finding is the strong correlation between tCBF in mL/min and both age and parenchymal volume. However, the association between tCBF and age was no longer found when flow was expressed in mL/100 mL/min. Second, when analysed separately, the flow in mL/min over each individual vessels contributing to the cerebral blood flow was associated with parenchymal volume as well. Third, when expressing tCBF in mL/100 mL/min a correlation between tCBF and total WMH volume was found. Fourth, no association was found between tCBF in mL/100 mL/min and atrophy.

In the present study we found a significant association between tCBF in mL/min and age, corresponding with an annual flow decrease of 6.2 mL. This confirms a previous study by Buijs et al. showing an average flow decrease of 4.8 mL/min per year[8]. The slightly higher decline of tCBF in mL/min with age found in our study is likely due to the fact that we included relatively older subjects and that all subjects had cardiovascular disease or risk factors for developing this condition. A strong association between tCBF in mL/min and parenchymal volume was found and remained after the adjustment for age. However, when the tCBF was expressed in mL/100 mL/min no significant association with age was found. Therefore it seems that parenchymal volume underlies the previous reported associations between age and tCBF mL/min.

In the present study the tCBF is obtained by the summation of the volume flow over the two carotid arteries and the two vertebral arteries. If the blood flow over these individual vessels is analysed separately, no association between the flow volume and age is found. This may be due to variation caused by the different conformations of the Circle of Willis as described by Hendrikse et al.[9] However, an association with parenchymal volume, the most important determinant of cerebral blood flow, was still present. Our data show a much higher association between the volume flow over internal carotid arteries and parenchymal volume than for the flow over the vertebral arteries. Both vertebral arteries contribute to the posterior circulation, which for a large part supplies the cerebellum. In this respect, it could be that parenchymal volume loss due to aging is different in the cerebellum than in the cerebrum.

Atherosclerotic patients have a relative high prevalence of WMHs and atrophy. In our population suffering from vascular pathology and cardiovascular riskfactors it was shown that tCBF in mL/100 mL/min was associated with WMH volume. No association was found between tCBF in mL/100 mL/min and atrophy. Although we can not draw a solid conclusion from our cross-sectional study, this finding indirectly suggests that blood flow to the brain probably plays a minor role in the development of atrophy. However, atrophy is best assessed using two time points, therefore the absence of associations between atrophy and tCBF should be replicated in a longitudinal study.

A potential limitation of our study is the small age range of the included patients. All patients in the present study were aged between 72 and 85 years, which makes extrapolation of our results to younger individuals arbitrary. On the other hand, WMHs and atrophy are highly prevalent and are thought to be clinically significant in this specific age span. Furthermore, the cross-sectional design of this study could not elucidate whether decreased tCBF precedes WMH or that reduced brain function due to the presence WMH reduces tCBF flow parameters.

### Conclusion

In conclusion, we found that tCBF in mL/min is strongly associated with the parenchymal volume rather than age. Although this finding seems obvious, this is the first study showing this strong association. In addition, we found a much weaker association between tCBF in mL/100 mL/min and the severity of WMHs. These findings have important implications for future studies in which flow measurements are being used as diagnostic tool: All future volume flow measurements should be expressed in mL/100 mL/min rather than mL/min. Moreover, when studying elderly patients or patients with a pathological increase of WMHs, such as diabetic type II subjects, tCBF measurements should also be corrected for WMH volumes.
